# Supplementary figures and images for: Metabolic and Molecular Rearrangements of Sauvignon Blanc (Vitis vinifera L.) Berries in Response to Foliar Applications of Specific Dry Yeast
Source: Plants (Basel). 2023 Sep 28;12(19):3423. doi: 10.3390/plants12193423 (PMC10574919; doi:10.3390/plants12193423)

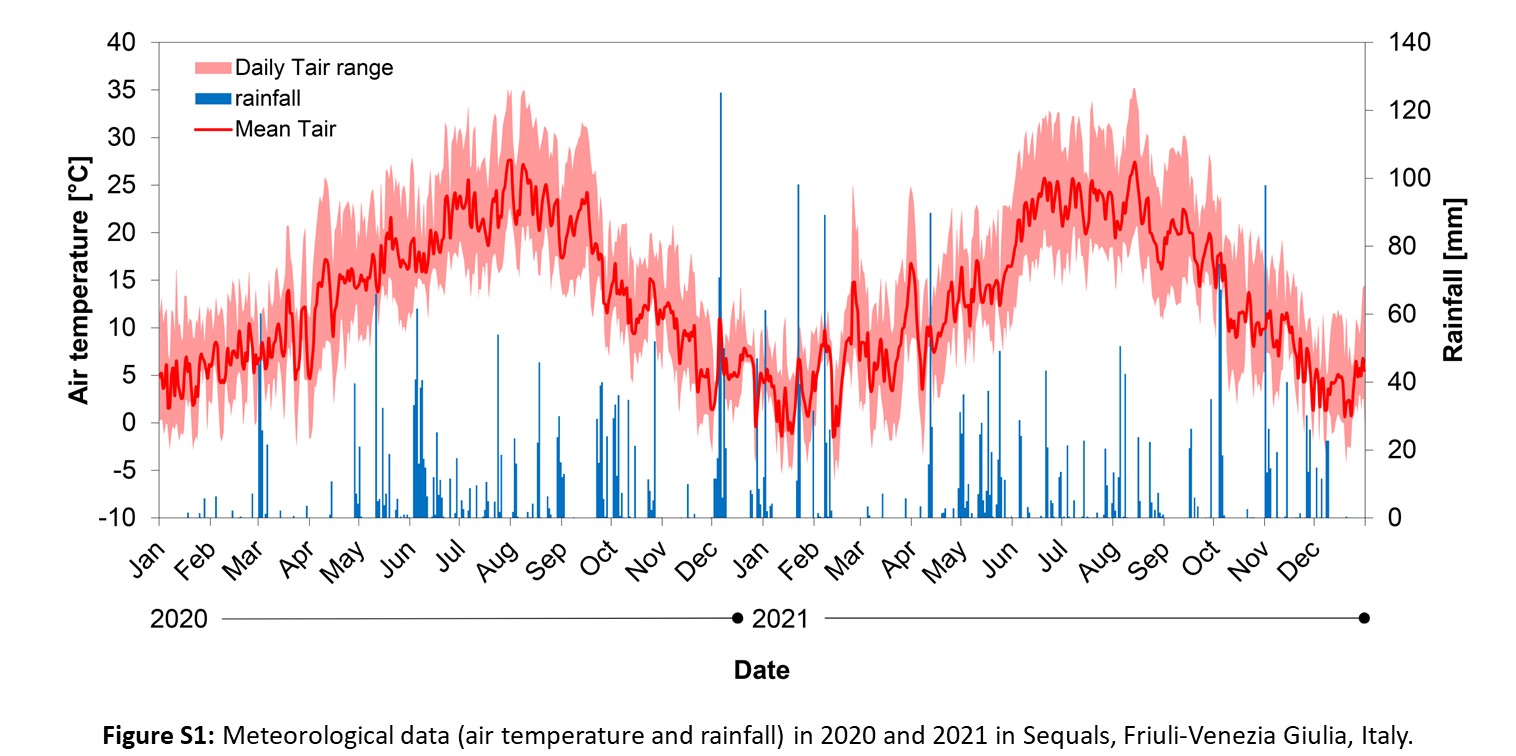

Supplement: Supplementary file 1 [file plants-12-03423-s001.zip › plants-2550638-Supplementary/Figure S1.jpg]
